# Performance of Two Trichogrammatid Species from Zambia on Fall Armyworm, *Spodoptera frugiperda* (J. E. Smith) (Lepidoptera: Noctuidae)

**DOI:** 10.3390/insects12100859

**Published:** 2021-09-23

**Authors:** Jia-Wei Sun, Hong-Ying Hu, Phillip O. Y. Nkunika, Peng Dai, Wei Xu, He-Ping Bao, Nicolas Desneux, Lian-Sheng Zang

**Affiliations:** 1 Institute of Biological Control, Jilin Agricultural University, Changchun 130118, China; sjw4501@163.com (J.-W.S.); xuwei1996@163.com (W.X.); 2 College of Life Science and Technology, Xinjiang University, Urumqi 830046, China; hoohyi-69@163.com; 3 Department of Biological Sciences, School of Natural Sciences, University of Zambia, 10101 Lusaka, Zambia; pnkunika@unza.zm; 4 Zambia Agricultural Technology Demonstration Centre, Jilin Agricultural University, 32379 Lusaka, Zambia; baohp2006@163.com; 5 Université Côte d’Azur, INRAE, CNRS, UMR ISA, 06000 Nice, France; nicolas.desneux@inrae.fr

**Keywords:** *Spodoptera frugiperda*, egg parasitoid, *Trichogramma mwanzai*, *Trichogrammatoidea lutea*, biological control agent

## Abstract

**Simple Summary:**

Two egg parasitoid species (*Trichogramma mwanzai* and *Trichogrammatoidea lutea*) from parasitized eggs of fall armyworm (FAW) *Spodoptera frugiperda* in Zambia were identified by using a combination of both molecular and morphological characters. We compared the parasitism capabilities of the two species with three native Chinese trichogrammatid species (*T. ostriniae*, *T. leucaniae* and *T. japonicum*) using 0- to 2-day-old FAW eggs. Both parasitoid species accepted eggs of all ages tested and completed their development successfully. *Trichogrammatoidea lutea* females showed the highest parasitism rate of host eggs among the five tested species. *T. mwanzai* had the shortest developmental time on all test age eggs. Of the five parasitoid species reared on FAW eggs, *T. lutea* performed the best overall, while *T. japonicum* was the worst performing of the parasitoids.

**Abstract:**

The fall armyworm, *Spodoptera frugiperda* (J.E. Smith), is a noctuid moth native to the tropical and subtropical Americas that has successfully invaded Africa and Asia, where it is has become a serious threat to food security as a pest of cereals and other crops. Biological control is an environmentally friendly means of combating the pest and contributes to an integrated pest management approach. In our study, two egg parasitoid species (*Trichogramma mwanzai* and *Trichogrammatoidea lutea*) found in parasitized fall armyworm eggs in Zambia were identified by using a combination of both molecular and morphological characteristics. To evaluate their potential and efficiency on 0- to 2-day-old fall armyworm eggs, we compared their parasitism capabilities with three *Trichogramma* species native to China (*T. ostriniae*, *T. leucaniae* and *T. japonicum*) under laboratory conditions. The results showed that both parasitoid species would accept 0-, 1- and 2-day-old fall armyworm eggs, and complete their development successfully. *Trichogramma mwanzai* and *T. lutea* preferred parasitizing 0- and 1-day-old eggs over 2-day-old eggs. *Trichogrammatoidea lutea* females supplied with fall armyworm eggs produced the highest parasitism rate of host eggs among the five tested species, while *T. mwanzai* had the shortest developmental time on all test age eggs. In general, *T. lutea* was the best performing of the five species when reared on fall armyworm eggs, while *T. japonicum* was the worst. There were no significant differences, however, in percent emergence in the five test species when reared on fall armyworm eggs.

## 1. Introduction

The fall armyworm, *Spodoptera frugiperda* (J.E. Smith) (=FAW), is a serious crop pest endemic to tropical and subtropical regions of the Americas. It is a species in the family Noctuidae, order Lepidoptera, and was first discovered on the African continent in 2016 [1,2]. It is a destructive pest that has been recorded from 353 host species belonging to 76 plant families, among which Poaceae, Fabaceae, Solanaceae, Asteraceae, Rosaceae, Chenopodiaceous, Brassicaceae and Cyperaceae are the most commonly affected [3,4]. Wide adaptability, strong migratory ability, high fecundity, lack of diapause, rapid development of resistance to insecticides, and voracious appetite have contributed to the difficulties encountered when attempting to manage this serious pest [5,6,7,8,9]. Recently, the species has broken out in Africa [2,10,11] and successfully invaded Europe, including Germany and The Netherlands [11], and has even reached several Asian countries including India, Myanmar, and China [12,13]. The infestation of FAW quickly spanned two continents (Europe and Asia) in just three years [13]. 

Absence of diapause and favorable climatic conditions in most African countries allow *S. frugiperda* to complete several generations per year wherever host plants, including offseason and irrigated crops, are available [9,14,15,16]. Based on estimates published by the Centre for Agriculture and Bioscience International (CABI), in the absence of natural enemies and proper control methods, FAW has the potential to cause maize yield losses of 8.3 to 20.6 t per year, in 12 African maize-producing countries [17]. These losses represent a range of 21–53% of the annual production of maize in the countries involved. In 2017, African countries experienced between USD 2.4 billion to USD 6.1 billion in crop loss due to this species. FAW has also been recorded on other important food crops in Africa including sorghum and millet [11,17,18,19]. 

The current management methods used to control FAW rely on intensive use of chemical insecticides, even though long-term insecticide application is known to lead to many serious problems in crops, notably pesticide resistance, negative impacts on non-targeted organisms and environmental degradation [20,21,22,23,24,25,26]. This damage has been responsible for serious problems throughout much of Africa, leading to excessive use of toxic insecticides and small-holder farmers using scientifically unproven methods, for example the application of ash, sand, botanical extracts, and other locally available materials [27]. According to available data, there are currently 121 worldwide species of parasitic wasps distributed in 10 families that can be used against FAW, including *Trichogramma pretiosum* (Riley), *Telenomus remus* (Nixon), *Campoletis sonorensis* (Cameron), *Cotesia marginiventris* (Cresson), and *Chelonus insularis* (Cresson) [28,29,30]. *Trichogramma* egg parasitoids (Hymenoptera: Trichogrammatidae) have been used worldwide as biological control agents because they can control insect pests at an early developmental stage, thereby preventing serious crop damage [31,32,33,34]. 

Although 12 parasitoid species are currently known to attack fall armyworm in Zambia, no egg parasitoids have been recorded to date [14]. Herein we report the finding of two egg parasitoids of FAW in samples from Zambia. The specific objectives of our study were to: (1) Identify both trichogrammatid species using a combination of external morphological characters including male external genitalia, and molecular analysis; (2) Explore the performance of both trichogrammatid species on FAW eggs of different ages; (3) Compare the bio-control potential of both parasitoid species from Zambia with three local trichogrammatid species (*T. ostriniae*, *T. japonicum*, and *T. leucaniae*) from China. The primary goal of this study was to determine the biological control potential of the above-mentioned parasitoids, and to provide a theoretical basis for their release in IPM programs involving FAW.

## 2. Materials and Methods 

### 2.1. Sample Collection and Population Establishment

Masses of fall armyworm (FAW) *Spodoptera frugiperda* (Smith) were collected from September to October 2019 from maize fields at the China-aid Zambia Agricultural Technology Demonstration Centre (15°21′30″ S, 28°27′27″ E), Lusaka, Zambia. FAW egg masses including parasitized and unparasitized eggs were separated, and each egg mass containing parasitized black eggs was placed into a glass tube (10 cm × 1.5 cm, length × diameter). Emerged parasitoid individuals were used to initiate a small-scale culture under laboratory conditions of 26 ± 1 °C, RH 70 ± 5% and a photoperiod of 14:10 (L:D) h. The parasitoid populations were raised on rice moth (*Corcyra cephalonica*) eggs and maintained at the above laboratory conditions. Every five generations, the population was rejuvenated by transferring in additional FAW eggs. In this study, three local Chinese *Trichogramma* species (*T. ostriniae*, *T. leucaniae* and *T. japonicum*) (which are often used as dominant parasitoids to control a variety of Lepidoptera insects) were also tested on the FAW eggs [34]. *Trichogramma* *ostriniae* and *T. leucaniae* were collected from parasitized eggs of *Leguminivora glycinivorella* (Matsumura) (Lepidoptera: Tortricidae) from Heihe (Heilongliang Prov.) (50.22° N, 127.53° E), and *T. chilonis* was collected from parasitized eggs of *Chilo suppressalis* (Walker) (Lepidoptera: Crambidae) from Changchun (Jilin Prov.) (43.89° N, 125.32° E) in northeastern China in 2011 [35]. These populations were also raised on *C. cephalonica* eggs, and maintained under laboratory conditions similar to the above. 

### 2.2. Morphological Identification

At least 50 parasitoid specimens were initially prepared on microscope slides using the method described by Noyes [36] with some modifications, as follows: prior to dissection, specimens were macerated in 5% KOH in a plastic dish (diameter = 2.0 cm) for 24 h, and then washed two to three times with distilled water. Male genitalia and wings were separated from other parts under a microscope and then dehydrated for 5 min each in 50, 75, 80, 85, 90, 95, and 100% graded alcohols. After allowing for evaporation of alcohol, specimens were fixed in Arabic gum for observation. 

Specimens were initially identified based on morphological characters using a combination of the following published taxonomic accounts: Schulten and Feijen [37,38], Lin [39], Nagaraja and Nagarkatti [40], Nagaraja [41,42], and Pinto [43,44,45]. 

### 2.3. Molecular Identification

At least 20 genomic DNA samples of trichogrammatid were extracted following Kumar et al. [46]. DNA was dissolved in 30 µL 1× TE buffer (10 mmol/L Tris-Cl, 1 mmol/L pH = 8.0 EDTA) and stored at −20 °C. The standard barcoding primer sequences of the cytochrome oxidase I (*COI*) used in this study were LCO1490 (5′-GGT CAA CAA ATC ATA AAG ATA TTG G-3′) and HCO2198 (5′-TAA ACT TCA GGG TGA CCA AAA AAT CA-3′) [47]. 

Polymerase chain reactions (PCR) were performed in a total volume of 20 µL, containing 6 µL 2× Taq Master Mix (containing 0.05 U/µL Taq DNA Polymerase, 2× Taq PCR buffer, 3 mM MgCl_2_ and 400 µM dNTP mix) (Sangon Biotech (Shanghai) Co., Ltd., Shanghai, China), 1.5 µL forward primer (10 µM), 1.5 µL reverse primer (10 µM), 2 µL genomic DNA (10–30 ng/µL), and 9 µL ddH_2_O. PCR amplifications were performed with denaturation at 94 °C for 5 min, followed by 35 cycles consisting of 94 °C for 30 s, 30 s at the primer-specific annealing temperature, 72 °C, for 30 s. The reactions were then kept at 72 °C for 10 min as the final step. PCR products were separated in 1× TAE buffer using a 1% agarose gel stained with ethidium bromide (EB), to confirm amplification. A 500 bp DNA ladder (Sangon Biotech (Shanghai) Co., Ltd., Shanghai, China) was used as a size marker. Qualified PCR products were sent to Sangon Biotech (Shanghai) Co., Ltd. for bidirectional sequencing, and compared to the *COI* sequences of known trichogrammatid species.

To further molecular biological identification, a total of 155 *COI* public sequences of *Trichogramma* sp. and *Trichogrammatoidea* sp. were downloaded, which represented records for the two species on the NCBI and the Bold System databases. We removed some of the sequences that were recorded from the same sample source; only 40 public trichogrammatid *COI* sequences were used to construct the phylogenetic tree NJ-tree analysis (Supplementary Table S1).

### 2.4. Assessment of Biological Characteristics

Based on the identification results obtained from the above procedures, the performance of the two African trichogrammatid species (*Trichogramma* sp. and *Trichogrammatoidea* sp.) and three local Chinese *Trichogramma* species (*T. ostriniae*, *T. leucaniae* and *T. japonicum*) were assessed when reared on FAW eggs of different ages. Preliminary observations showed that under laboratory conditions FAW eggs hatched within four days. Thus, 0-, 1- and 2-day-old host eggs were selected for the study involving the five trichogrammatid species.

The experiment was conducted under laboratory conditions at 26 ± 1 °C, RH of 70 ± 5%, and a photoperiod of 14:10 (L:D) h. One newly emerged (<8 h old), mated, female parasitoid was introduced into a glass tube with one 0-, 1- or 2-day-old egg mass containing 100 to 120 eggs. The female adults were allowed to oviposit for 24 h. Tests involving individuals that died or were subsequently lost during the experimental period were considered invalid. To avoid host larvae feeding on parasitized eggs, the larvae emerging from host eggs were removed by brush as soon as they hatched. The number of parasitized eggs was recorded under a stereoscopic microscope eight days after the parasitoids were removed. The eggs were checked daily to determine the emergence of parasitoid offspring until no further wasps emerged. For each treatment, the number of parasitized eggs (number of the host eggs parasitized in black), the developmental time (d) (the number of days from exposure of host eggs to the parasitoid to the adult offspring emerging from host egg), percent emergence (the number of parasitized eggs with emergence holes/total number of parasitized eggs × 100), and percent female progeny (number of emerged females/total number of emerged females and males × 100) were recorded. There were 15 replicates for each species tested and for each of the egg ages tested. 

### 2.5. Statistical Analysis

#### 2.5.1. Phylogenetic Analysis 

Overall, sequences obtained in this study and similar sequences retrieved from GenBank were aligned using ClustalW implemented in MEGA 6.0 software [48]. The *COI* barcode relationships of all sequences were inferred from the reference sequences using BLAST tool in the GenBank database and Bold Systems database. Initial tree(s) for the heuristic search were obtained automatically by applying neighbor-join and BioNJ algorithms to a matrix of pairwise distances estimated using the Maximum Composite Likelihood (MCL) approach, and then selecting the topology with superior log likelihood value. The tree was drawn to scale, with branch lengths measured in the number of substitutions per site. Codon positions included were 1st + 2nd + 3rd + Noncoding. Branch support was assessed via bootstrapping with 1000 replicates. All positions containing gaps and missing data were eliminated. 

#### 2.5.2. Bioassay Analysis 

The data sets obtained in each bioassay regarding the number of eggs parasitized, percent emergence, developmental time and percent female progeny were analyzed by a linear model with parasitoid species (5 levels) and host egg age (3 levels) as factors using Tukey’s honest significant difference (HSD) test at *p* < 0.05. Before a GLM was performed, log transformation of number of eggs parasitized was applied, percent emergence and percent female progeny were arcsine square-root-transformed to homogenize variances and were subjected to the Shapiro–Wilk test. All statistical analyses were performed using the SPSS version 20 software package (SPSS Inc., Chicago, IL, USA).

## 3. Results

### 3.1. Morphological Identification 

**Figure 1:**
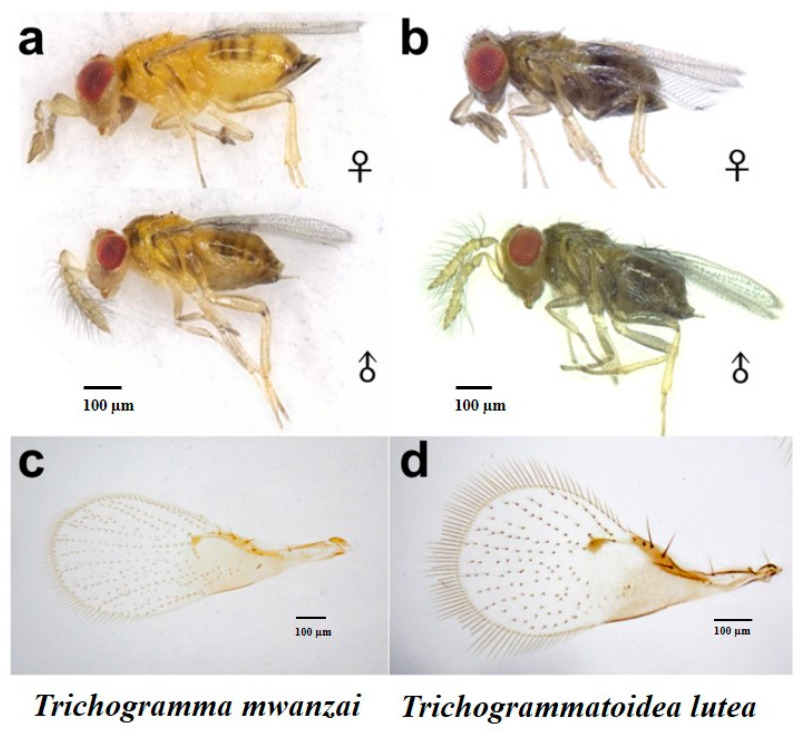
Comparison of adult morphological characters of two trichogrammatid species. ((**a**): male and female of *Trichogramma mwanzai*; (**b**): male and female of *Trichogrammatoidea lutea*; (**c**): forewing of *T. mwanzai*; (**d**): forewing of *T. lutea*).

The two trichogrammatid species from Zambia were initially identified as *Trichogramma mwanzai* (Figure 1a) and *Trichogrammatoidea lutea* (Figure 1b) based on morphological characters including male genital capsules. 

### 3.2. Molecular Biological Identification Based on COI

**Figure 2:**
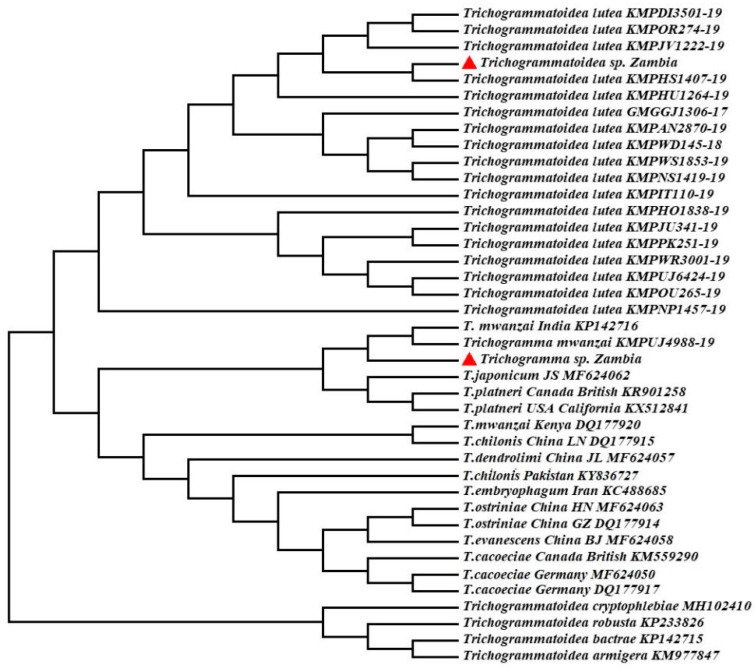
Molecular phylogenetic tree construction of *Trichogramma* and *Trichogrammatoidea* species based on the neighbor-joining method.

The sequencing of the *COI* gene of *Trichogramma* sp. and *Trichogrammatoidea* sp. resulted in two sequences, a 708 bp fragment of *Trichogramma* sp. (Accession number: MZ7711322) and 716 bp fragment of *Trichogrammatoidea* sp. (Accession number: MZ7711343). The results of the search for similarities by BLAST tool in the NCBI database and Bold Systems database verified that they were the targeted sequences. The results of blast showed that the *COI* sequences of *Trichogramma* sp. from Zambia showed 96.30% average identity with published sequences from *T. mwanzai* in India (Accession number: KP142716). The *COI* sequences of *Trichogrammatoidea* sp. shared over 98.0% homology identity with the sequences of *T.* *lutea* from South Africa (Accession number: KMPOU265-19). Moreover, NJ-tree (Figure 2) showed that *Trichogramma* sp. was close to *Trichogramma mwanzai*, and *Trichogrammatoidea* sp. was nested into the *Trichogrammatoidea lutea* cluster. 

The results of molecular biological identification, in combination with morphological evidence, indicated that *Trichogramma* sp. and *Trichogrammatoidea* sp. were conspecific with *Trichogramma mawanzai* and *Trichogrammatoidea lutea*, respectively. 

### 3.3. Performance on Host Eggs

Host age, parasitoid species, and the interaction between these two factors all showed significant effects on the parasitism number, developmental time, and percentage of female progeny of the five egg parasitoid species. There was no effect on the percentage of adult emergence (Table 1).

#### 3.3.1. Parasitism of *Trichogramma* and *Trichogrammatoidea* Species on Different Ages of FAW Eggs

**Figure 3:**
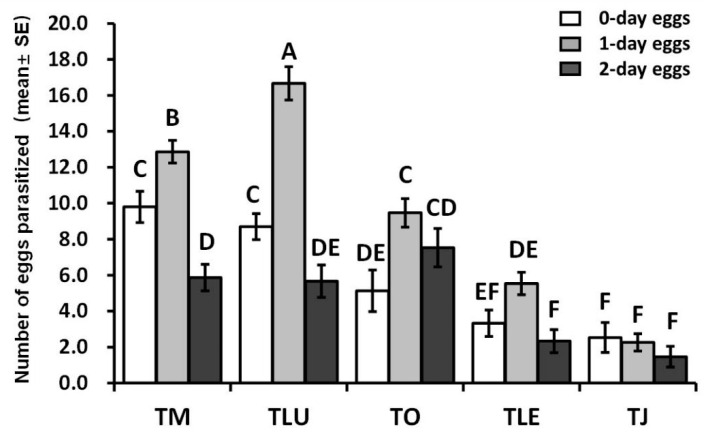
Mean number of FAW eggs parasitized by the five parasitoid species within a 24 h period at various host egg ages. (Different upper-case letters above group bars indicate significant difference (*p* < 0.05, one way ANOVA followed by Tukey’s test) in number of parasitized eggs among the five species using eggs of different ages.). TM: *T. mwanzai*; TLU: *T. lutea*; TO: *T. ostriniae*; TLE: *T. leucaniae*; TJ: *T. japonicum*.

The results showed that all five species parasitized FAW eggs (Figure 3). The egg age of FAW had a significant impact on the number of eggs parasitized by *T. mwanzai* (*F*_2,42_ = 21.797, *p* < 0.0001), *T. lutea* (*F*_2,42_ = 39.07, *p* < 0.0001), *T. ostriniae* (*F*_2,42_ = 4.531, *p* = 0.017), and *T. leucaniae* (*F*_2,42_ = 6.021, *p* = 0.005). With the exception of *T. japonicum*, all trichogrammatid species preferred to parasitize 1-day-old eggs over 0-, and 2-day-old eggs. *Trichogrammatoidea lutea* and *T. mwanzai* parasitized a significantly higher number of 0-day-old eggs (*F*_4,70_ = 12.652, *p* < 0.0001) and 1-day-old eggs (*F*_4,70_ = 64.776, *p* < 0.0001) than the other three species. *T. lutea* produced the highest number of parasitized eggs (16.7) on 1-day-old eggs among the five tested species (*F*_4,70_ = 64.776, *p* < 0.0001), followed by *T. mwanzai*, *T. ostriniae*, and *T. leucaniae*, and *T. japonicum*, which parasitized the fewest hosts in all the egg ages. All of the species studied failed to parasitize more than 20 FAW eggs in a 24 h period, although more than 100 host eggs were provided in every instance.

#### 3.3.2. Development of *Trichogramma* and *Trichogrammatoidea* Species on FAW Eggs of Different Ages

The developmental time of the five tested species was significantly different on eggs of different ages (0-day-old: *F*_4,55_ = 10.412, *p* < 0.0001; 1-day-old: *F*_4,70_ = 27.830, *p* < 0.0001; 2-day-old: *F*_4,63_ = 22.690, *p* < 0.0001). *Trichogramma* *ostriniae* had a similar developmental time on different ages of host eggs (*F*_2,42_ = 1.531, *p* = 0.228), whereas the developmental time of *T. leucaniae* (*F*_2,35_ = 14.155; *p* < 0.0001) and *T. japonicum* (*F*_2,42_ = 6.964, *p* = 0.003) increased as the age of the host eggs increased (Table 2). Conversely, the developmental time of *T. mwanzai* (*F*_2,42_ = 22.879, *p* < 0.0001) and *T. lutea* (*F*_2,42_ = 29.682, *p* < 0.0001) showed a tendency to decrease as the age of the host egg increased. *Trichogrammatoidea lutea* had a distinct prolonged developmental time when reared on 0-day-old eggs, while *T. mwanzai* had the shortest developmental time on 1-, and 2-day-old eggs among all species.

#### 3.3.3. Emergence and Sex Ratio of *Trichogramma* and *Trichogrammatoidea* Species Reared on FAW Eggs of Different Ages

There were no significant differences in the percent emergence of the five tested species on various egg ages of FAW (0-day-old egg: *F*_4,61_ = 0.585, *p* = 0.675; 1-day-old egg: *F*_4,70_ = 0.922, *p* = 0.456; 2-day-old egg: *F*_4,57_ = 1.276, *p* = 0.290) (Table 2). Significant differences were found in the percent female progeny of *T. mwanzai* (*F*_2,42_ = 20.908, *p* < 0.0001) and *T. lutea* (*F*_2,42_ = 38.181, *p* < 0.0001) on 0-, 1- and 2-day-old eggs (Table 2). *Trichogramma* *japonicum* and *T. lutea* had the lowest percent female progeny on 1-day-old eggs (*F*_4,70_ = 6.336, *p* < 0.0001) and 2-day-old eggs (*F*_4,61_ = 2.792, *p* = 0.034), respectively, among all tested species. There was no significant difference in the percentage of female progeny of *T. ostriniae* or *T. leucaniae* among all tested ages.

## 4. Discussion

*Trichogramma* and *Trichogrammatoidea* species reared from parasitized eggs of *Spodoptera frugiperda* in Zambia were identified based primarily on genitalic and genetic characteristics. The COI sequence of *Trichogramma sp.* was similar to *Trichogramma mwanzai* [38] which have been discovered in rice fields in Malawi (Central Africa), and also reported from maize fields in Kenya [38]. Previous reports indicate that *T. mwanzai* could potentially be used against the eggs of several lepidopterous pests in Africa, such as *Chilo partellus* Swinhae, *Busseola fusca* (Fuller), *Helicoverpa armigera* (Hübner), and *Plutella xylostella* (L.) [49,50,51]. COI sequencing of *Trichogrammatoidea* sp. was similar to *Trichogrammatoidea lutea* described by Nagaraja in his studies on the genus [42]. Most of the lepidopteran hosts were reported to be attacked by *T.* sp. nr. *lutea* in eastern Africa [52], and *T. lutea* in South Africa [53]. Recently, *T. lutea* has also been found parasitizing FAW eggs in Sadore, Niger [54].

The parasitism and host preference of the egg parasitoids are often determined by the species of parasitoids [55,56,57]. In our study, all species accepted and attacked the FAW egg masses, although *T. mwanzai* and *T. lutea* parasitized significantly more FAW eggs (of all ages) than *T. japonicum*, *T. ostriniae*, and *T. leucaniae*. *Trichogramma mwanzai* and *T. lutea* parasitized significantly higher numbers of 0-, and 1-day-old eggs than older eggs, suggesting that they preferred these over the older host eggs. Usually, moth scales covering FAW egg masses are quite common and most of the test eggs were covered by scales, constituting a barrier against parasitization [55,58]. Therefore, it is not surprising that all tested trichogrammatid species parasitized fewer than 20% of the FAW eggs provided, with most of the parasitized eggs being those that were not covered by the scales. However, the effectiveness of parasitism by *T. mwanzai* and *T. lutea* is quite low compared to *Telenomus remus* Nixon (Hymenoptera: Platygastridae), a parasitoid native to the Malay Peninsula that has been released in biological control programs against FAW in the Americas [59,60]. In a recent study, *T. remus* was shown to parasitize more FAW eggs than *Trichogrammatoidea* sp. due to its ability to overcome the layer of scales on the egg masses [54]. This is consistent with previous findings [61]. Similar results were obtained in another species of *Trichogramma, T. pretiosum,* on eggs of the closely related noctuid host species, *Spodoptera frugiperda* and *S. litura* [57]. Differences in parasitism among species may also be attributed to physical stress of the host, which may affect the oocyte, resulting in increased permeability of the egg [62]. Increased mortality of immature trichogrammatids reported by Calvin et al. [63] and Lund [64] was attributed to decreasing humidity associated with hardening of the eggshells. The relationship between eggshell structure and resistance to desiccation has been studied in several Lepidoptera species [65].

Host age may influence the preference of some parasitoids [66,67,68] and the response to host age is independent of the egg parasitoid species [69]. We observed that, although *T. mwanzai* and *T. lutea* accepted all of the host eggs at various ages, in all cases the older host eggs, i.e., 2-day-old, were significantly less parasitized than 0-day-old and 1-day-old eggs. It is common among egg parasitoids that parasitism decreases with increasing host age [68,70,71,72]. For example, *T. chilonis* showed a strong preference for 1–2-day-old eggs of *P. xylostella* [73] or 0-day-old eggs of *C. suppressalis* [72]. *Trichogramma ostriniae* preferred to parasitize younger eggs of *Mythimna separata* (Walker) (Lepidoptera: Noctuidae) at all egg ages, but no significant difference was observed in our case [35]. The rejection of older eggs could be attributed to the change of color, hardening of the chorion, rotation of the host embryo or the sclerotization of the head capsule, making it difficult for the parasitoid to probe the egg [67,74]. In addition, *T. japonicum* and *T. leucaniae* tended to have prolonged developmental time on older eggs than on the younger ones (Table 2). Similar observations were reported by Hou et al. [35], who indicated that the development of *T. ostriniae* was significantly slower with increase in age and that no *T. japonicum* parasitoids emerged from 3-day-old eggs of *M. separata*. The possible mechanism underlying these phenomena is the difference in host quality associated with host egg age and the increasing defense capacity with the development of the host egg [75]. In contrast, the developmental time of *T. mwanzai* and *T. lutea* in our study showed a tendency to decrease with increasing age. This suggests that host selection and adaptation for some parasitoids could be influenced by the interaction between host age and species [66,67,72]. The *Trichogramma* species in previous studies exhibit different acceptance for host eggs at different ages [68]. Several previous studies showed that the first 75% of embryological development of the host egg was suitable for trichogrammatid larval development, although the pattern of vulnerability with host age varied among species [67,76].

When the biological control agent is a polyphagous species, it is quite useful to confirm host suitability and parasitism through the test traits (i.e., egg parasitism, adult emergence rate, female progeny, and developmental time) with more than one host species [67,72,77,78]. For example, *T. ostriniae* from Taiwan, China almost completely ignored *Ostrinia nubilalis* (Hb.) eggs and only parasitized *Sitotroga cerealella* (Olivier) eggs [79]. In Kenya, *T. mwanzai* exhibited a positive preference for *Chilo partellus* Swinhae eggs over other native host species, but eggs of the silkworm *Bombyx mori* L. were not attacked [80]. Similarly, in our study, *T. mwanzai* and *T. lutea* parasitized and successfully developed in all FAW eggs tested. Recently, we also found that both trichogrammatid species could be effectively produced on the alternative host, *Corcyra cephalonica* (Stainton) eggs (unpublished data). In general, the ability to control FAW is superior in *T. lutea* than it is in the other tested species. Nevertheless, our results indicate that the *Trichogrammatoidea* species show potential for use in biological control programs directed against the FAW in Africa.

## 5. Conclusions

We found and identified two species of parasitoids that can be used to potentially control FAW eggs in the field. Both of the parasitoids showed significantly higher parasitism rates on FAW at various egg ages than the three tested species from China. This finding lays the groundwork for the use of *T. lutea* or *T. mwanzai* in biocontrol programs in integrated management of the FAW in Africa and China. Likewise, the rearing of *T. mwanzai* and *T. lutea* on an alternative host such as *Corcyra cephalonica* Stainton, should be considered for cost-effective mass production.

## Figures and Tables

**Table 1:** Summary of the ANOVA analysis on effects of host age and parasitoid species on the number of eggs parasitized, percentage of adult emergence, developmental time, and percentage of female progeny of the five parasitoid species.

Traits	Variance Source	Df	*F*	*p*
Number of eggs parasitized	Host age	2	46.310	<0.001
	Parasitoid species	4	58.908	<0.001
	Parasitoid species × host age	8	8.243	<0.001
	Error	210	-	-
Percent adult emergence	Host age	2	1.698	0.186
	Parasitoid species	4	1.692	0.154
	Parasitoid species × host age	8	0.773	0.627
	Error	188	-	-
Developmental time	Host age	2	3.479	0.033
	Parasitoid species	4	4.433	0.002
	Parasitoid species × host age	8	11.447	<0.001
	Error	188	-	-
Percent female progeny	Host age	2	4.420	<0.001
	Parasitoid species	4	17.230	0.025
	Parasitoid species × host age	8	2.890	0.070
	Error	188	-	-

**Table 2:** Comparisons of developmental time, percent emergence, and percent female progeny of the five parasitoid species on FAW eggs of different ages.

Parameters	Species	No. of Egg Masses	Host Age (Days)		
			0	1	2
Developmental time (days)	*T. mwanzai*	15	10.5 ± 0.1 b A	9.5 ± 0.1 d B	9.9 ± 0.1 b B
	*T. lutea*	15	11.4 ± 0.1 a A	10.5 ± 0.1 bc B	10.2 ± 0.1 b B
	*T. ostriniae*	15	10.5 ± 0.2 b A	10.2 ± 0.2 c A	10.5 ± 0.2 b A
	*T. leucaniae*	15	10.3 ± 0.2 b B	11.6 ± 0.2 a A	11.4 ± 0.2 a A
	*T. japonicum*	15	10.6 ± 0.2 b B	11.0±0.2a b AB	11.7 ± 0.2 a A
% Emergence	*T. mwanzai*	15	98.3 ± 0.9 a A	94.8 ± 2.0 a A	93.2 ± 2.1 a A
	*T. lutea*	15	98.7 ± 0.9 a A	99.4 ± 0.3 a A	98.2 ± 1.4 a A
	*T. ostriniae*	15	96.4 ± 1.8 a A	93.3 ± 2.6 a A	97.5 ± 1.2 a A
	*T. leucaniae*	15	96.7 ± 2.6 a A	96.3 ± 2.4 a A	97.1 ± 1.9 a A
	*T. japonicum*	15	99.1 ± 0.9 a A	90.2 ± 6.9 a A	85.7 ± 14.3 a A
% Female progeny	*T. mwanzai*	15	85.1 ± 2.2 a A	82.3 ± 2.8 a A	60.1 ± 3.8 a B
	*T. lutea*	15	82.2 ± 3.7 a A	79.7 ± 2.7a A	50.8 ± 1.6 b B
	*T. ostriniae*	15	82.5 ± 5.6 a A	77.2 ± 3.6 a A	69.9 ± 4.1 a A
	*T. leucaniae*	15	82.7 ± 5.9 a A	77.6 ± 5.0 a A	70.8 ± 7.1 a A
	*T. japonicum*	15	63.6 ± 13.2 a A	49.6 ± 9.4 b A	64.4 ± 11.8 a A

Note: Mean ± SE are presented. Means in a column followed by the same lowercase letter and means in a row followed by the same uppercase letter do not differ significantly (*p* < 0.05) using Tukey’s HSD test.

## Data Availability

The data presented in this study are available in article and Supplementary Materials.

## Supplementary Materials

The following are available online at https://www.mdpi.com/article/10.3390/insects12100859/s1, Table S1: COI sequences of trichogrammatid species used in this study.
