# Supplementary material for: Performance of Two Trichogrammatid Species from Zambia on Fall Armyworm, Spodoptera frugiperda (J. E. Smith) (Lepidoptera: Noctuidae)
Source: Insects. 2021 Sep 23;12(10):859. doi: 10.3390/insects12100859 (PMC8538588; doi:10.3390/insects12100859)
Supplement: Supplementary file 1 [file insects-12-00859-s001.zip › insects-1355860-supplementary.pdf]

Supplementary Table S1. *COI* sequences of trichogrammatid species used in this study

Species	COI-Product	Origin	Accession number	Database
Trichogramma cacoeciae	607 bp	Canada British Columbia	KM559290	NCBI
Trichogramma cacoeciae	518 bp	Germany	MF624050	NCBI
Trichogramma cacoeciae	518 bp	Germany	DQ177917	NCBI
Trichogramma chilonis	518 bp	China Liaoning	DQ177915	NCBI
Trichogramma chilonis	627 bp	Pakistan: Islamabad	KY836727	NCBI
Trichogramma dendrolimi	518 bp	China Jilin	MF624057	NCBI
Trichogramma embryophagum	684 bp	Iran	KC488685	NCBI
Trichogramma evanescens	518 bp	China Beijing	MF624058	NCBI
Trichogramma japonicum	518 bp	China Jiangsu	MF624062	NCBI
Trichogramma mwanzai	654 bp	India	KP142716	NCBI
Trichogramma mwanzai	518 bp	Kenya	DQ177920	NCBI
Trichogramma mwanzai	647 bp	South Africa Mpumalanga	KMPUJ4988-19	BOLD Systems
Trichogramma ostriniae	518 bp	China Guangzhou	DQ177914	NCBI
Trichogramma ostriniae	518 bp	China Hainan	MF624063	NCBI
Trichogramma platneri	652 bp	USA California	KX512841	NCBI
Trichogramma platneri	570 bp	Canada British Columbia	KR901258	NCBI
Trichogramma sp.	471 bp	Zambia Lusaka	MZ7711322	NCBI
Trichogrammatoidea armigera	489 bp	India	KM977847	NCBI
Trichogrammatoidea bactrae	642 bp	India	KP142715	NCBI

Trichogrammatoidea cryptophlebiae	617 bp	Israel	MH102410	NCBI
Trichogrammatoidea lutea	472 bp	Gabon Moyen-Ogooue	GMGGJ1306-17	BOLD Systems
Trichogrammatoidea lutea	640 bp	South Africa Mpumalanga	KMPAN2870-19	BOLD Systems
Trichogrammatoidea lutea	641 bp	South Africa Mpumalanga	KMPDI3501-19	BOLD Systems
Trichogrammatoidea lutea	640 bp	South Africa Mpumalanga	KMPHO1838-19	BOLD Systems
Trichogrammatoidea lutea	646 bp	South Africa Mpumalanga	KMPHS1407-19	BOLD Systems
Trichogrammatoidea lutea	649 bp	South Africa Mpumalanga	KMPHU1264-19	BOLD Systems
Trichogrammatoidea lutea	648 bp	South Africa Mpumalanga	KMPIT110-19	BOLD Systems
Trichogrammatoidea lutea	641 bp	South Africa Limpopo	KMPJU341-19	BOLD Systems
Trichogrammatoidea lutea	647 bp	South Africa Limpopo	KMPJV1222-19	BOLD Systems
Trichogrammatoidea lutea	648 bp	South Africa Mpumalanga	KMPUJ6424-19	BOLD Systems
Trichogrammatoidea lutea	671 bp	South Africa Limpopo	KMPWD145-18	BOLD Systems
Trichogrammatoidea lutea	645 bp	South Africa Mpumalanga	KMPWR3001-19	BOLD Systems
Trichogrammatoidea lutea	645 bp	South Africa Mpumalanga	KMPWS1853-19	BOLD Systems
Trichogrammatoidea lutea	652 bp	South Africa Limpopo	KMPNP1457-19	BOLD Systems
Trichogrammatoidea lutea	648 bp	South Africa Limpopo	KMPNS1419-19	BOLD Systems
Trichogrammatoidea lutea	638 bp	South Africa Limpopo	KMPOR274-19	BOLD Systems
Trichogrammatoidea lutea	649 bp	South Africa Limpopo	KMPOU265-19	BOLD Systems
Trichogrammatoidea lutea	640 bp	South Africa Limpopo	KMPPK251-19	BOLD Systems
Trichogrammatoidea robusta	617 bp	India	KP233826	NCBI
Trichogrammatoidea sp.	471 bp	Zambia Lusaka	MZ7711343	NCBI
